# Sex differences in presentation of atrial fibrillation: Findings from 30-day ambulatory monitoring in real-world practice

**DOI:** 10.1016/j.ahjo.2022.100208

**Published:** 2022-09-16

**Authors:** Jian Liang Tan, Linda Johnson, Marek Dziubinski, Natan Napiorkowski, Olga Witkowska, Magdalena E. Slusarczyk, Jeff S. Healey, Andrea M. Russo

**Affiliations:** a Cardiovascular Division, Cooper University Health System, Cooper Medical School of Rowan University, United States of America; b Department of Clinical Sciences, Malmö, Lund University, Lund, Sweden; c Medicalgorithmics S. A., Warsaw, Poland; d Population Health Research Institute, McMaster University, Hamilton, Ontario, Canada

**Keywords:** Sex differences, Atrial fibrillation, Mobile cardiac telemetry, Arrhythmia

## Abstract

**Background:**

Women are less likely to receive oral anticoagulation or ablation for treatment of atrial fibrillation (AF). Identification of sex differences in arrhythmia characteristics and symptoms may lead to a better understanding of potential reasons for these differences.

**Objectives:**

To determine sex differences in AF with respect to heart rate, duration, burden, and symptoms in patients undergoing mobile cardiac telemetry (MCT) monitoring.

**Methods:**

All patients who registered for ≤30-day MCT using PocketECG (MediLynx) in the USA in 2017 were included (n = 27,512, 58 % women). PocketECG records and transmits a three-lead ambulatory electrocardiogram (ECG) with real-time beat-to-beat analysis. Sex-related differences were analyzed with Chi2 and Spearmans rho.

**Results:**

Fewer women than men were diagnosed with AF lasting ≥30s (13.7 % versus [vs] 19.0 %, p < 0.001). AF burden was lower in women in all age groups <90 years (all p < 0.01). Women were older at the time of AF diagnosis (median 76 vs 73 years, p < 0.001), had faster heart rate during AF (mean: 104.7 ± 26.0 vs 96.7 ± 26.7 bpm, p < 0.001), and shorter AF duration (mean: 96.2 ± 176.0 vs 121.6 ± 189.9 min, p < 0.001). There was a non-significant trend toward more symptoms (such as dizziness, racing heart, fatigue, or palpitations) during AF in women compared to men (46.5 % vs 43.7 %, p = 0.062).

**Conclusions:**

AF was less prevalent and occurred at lower burdens in women than men in each age strata. Despite faster heart rates in AF in women, there were no significant sex differences in reported symptoms during AF. Sex differences in therapy cannot be explained by differences in symptoms or rates in AF.

**Condensed abstract:**

Real-world data on sex differences in AF using a 30-day MCT monitoring device remain scarce. We aim to determine the sex differences in AF with respect to prevalence, burden, heart rate, and symptom in patients undergoing ≤30-day MCT monitoring. Our data analysis suggests that fewer women than men had AF, women were older at diagnosis of AF, and women with AF had higher mean heart rate, shorter mean AF duration, and lower mean AF burden than men. Further studies are needed to examine reasons for sex differences, specifically in relation to AF therapy and its impact on clinical outcomes.

## Graphical abstract

**Figure:**
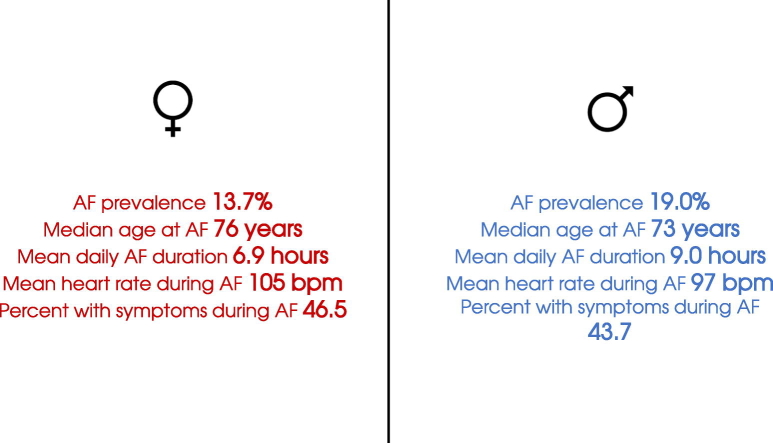

## 1 Introduction

Atrial fibrillation (AF), one the most prevalent sustained tachyarrhythmia encountered in clinical practice, is associated with considerable morbidity and mortality [1]. It affects women and men differently, including symptoms and AF burden with associated differences in clinical management [2]. Female sex is a known independent risk modifier for ischemic stroke in patients with AF [3]. In fact, the risk of stroke among women with AF, especially aged ≥75 years, is higher than men in those with ≥2 non-sex-related stroke risk factors [4]. As such, female sex has been incorporated into the AF risk score and clinical guidelines for thromboembolic risk assessment [4].

It has been shown in several studies that women with AF report higher symptom burden compared to men [5], [6]. However, women are less likely to receive oral anticoagulation or to be offered AF-related interventions (i.e. electric cardioversion, left atrial appendage closure, or catheter ablation) [5], [6], [7]. Women were under-represented in some major clinical trials (i.e., EARLY-AF [Early Aggressive Invasive Intervention for Atrial Fibrillation], PROTECT AF [WATCHMAN Left Atrial Appendage System for Embolic PROTECTion in Patients With Atrial Fibrillation], PINNACLE FLX study [Protection Against Embolism for Nonvalvular AF Patients: Investigational Device Evaluation of the Watchman FLX LAA Closure Technology]) evaluating therapies for treatment of AF [8], [9], [10], [11]. Hence, clinical application of the sex-specific analyses from those trials remains challenging and limited.

We aim to examine sex differences in AF with respect to prevalence, burden, heart rate and symptoms during 30-day ambulatory rhythm monitoring using mobile cardiac telemetry (MCT) monitors (PocketECG, MediLynx). We hypothesized that the presentation of AF differs in women and men with respect to heart rate, duration, burden, and symptoms, across all age groups.

## 2 Methods

### 2.1 Mobile telemetry system

The MediLynx PocketECG monitor is Food and Drug Administration-approved for outpatient monitoring of cardiac arrhythmias. It records and transmits full-disclosure three-lead ECGs continuously, with near-real time assessment of cardiac rhythm. All beats are labeled using a proprietary algorithm and manual over-read by ECG technicians. Symptoms can be reported using the device by button click and drop-down menu characterization, including symptoms such as dizziness, racing heart, fatigue, fluttering or palpitations. In this study, reported symptoms have been matched with arrhythmias detected on ECG signals during or shortly prior to (within 30 s) reported symptoms. If a symptom was reported during an episode of AF, it would be labeled as an “AF-related symptom.” In contrast, if a symptom was reported despite the lack of an arrhythmia on the ECG recording, such symptom would be labeled as a sinus rhythm-related symptom. All the transmitted information and ECG data are stored securely in a database by MediLynx.

### 2.2 Data sources, definitions and study population

We performed a retrospective analysis of the ECG data collected and stored in the MediLynx database. The MCT monitors were prescribed by the providers for a variety of indications based on the International Classification of Diseases, 10th Revision, Clinical Modification (ICD-10-CM) diagnosis codes. Patients who had registered for up to 30-day ECGs recordings using the MediLynx Pocket ECG monitors in the year 2017 (27,512 patients) were included in this study.

An AF event was defined as an arrhythmia without p-waves and a duration of ≥30 s. Average heart rates during AF were recorded. Age, sex, reported symptoms, heart rate, and AF burden were also examined.

The Committee on Human Research at Cooper University Hospital deemed that this study did not meet the definition of human subjects' research. Hence, this study received exemption from the institutional review board review.

### 2.3 Statistical analysis

Normally distributed continuous variables are reported as means and standard deviations (SD), and skewed variables are reported as medians (inter-quartile range). Categorical variables are reported as percentages (n, %). Sex differences were analyzed using the Wilcoxon rank-sum tests for the continuous variables (such as age, heart rate or duration of AF) and the Pearson chi-squared test for the categorical variables. The Spearman's Rank correlation was used to measure the degree of association between two numerical variables (such as AF burden and age) in both women and men. Two-way ANOVA was used to explore possible interaction between AF burden and sex. Statistical analyses were performed using Python scripting language in Anaconda environment and libraries Pandas, Numpy, and Scipy. All the tests were 2-sided and a p value <0.05 was considered statistically significant.

## 3 Results

Of 27,512 patients who underwent MCT monitoring for up to 30-days, 15,953 patients (58.0 %) were female and 11,559 (42.0 %) were male. Table 1 depicts the clinical characteristics of patients who wore MCT monitors for up to 30-days stratified by sex. The mean age was 67.4 ± 16.1 years in women and 68.6 ± 14.5 years in men (p < 0.001).

**Table 1:** Baseline characteristics, AF frequency and symptoms by sex.

Variables	Overall	Women	Men	p value
Number of patients with 30-day MCT monitors, n (%)	27,512	15,953 (58.0)	11,559 (42.0)	<0.001
Age, mean ± SD, years	68.2 ± 15.0	67.4 ± 16.1	68.6 ± 14.5	<0.001
AF recorded, n (%)				<0.001
Yes	4379 (15.9)	2180 (13.7)	2199 (19.0)	
No	23,133 (84.1)	13,773 (86.3)	9360 (81.0)	
Symptoms during monitoring, n (%)				<0.001
Yes	18,811 (68.4)	11,387 (71.4)	7424 (64.2)	
No	8701 (31.6)	4566 (28.6)	4135 (35.8)	

AF events were found in 4379 patients (15.9 %). An AF event was found in 2180 (13.7 %) women and 2199 (19.0 %) men (*X*^2^ = 143.4, p < 0.001). The majority of the patients (n = 18,881, 68.4 %) reported a symptom of some type at least once. Compared with men (64.2 %), women (71.4 %) were more likely to report a symptom of some type for at least once during the 30-day period of MCT monitoring (*X*^2^ = 158.2, p < 0.001).

**Fig. 1:**
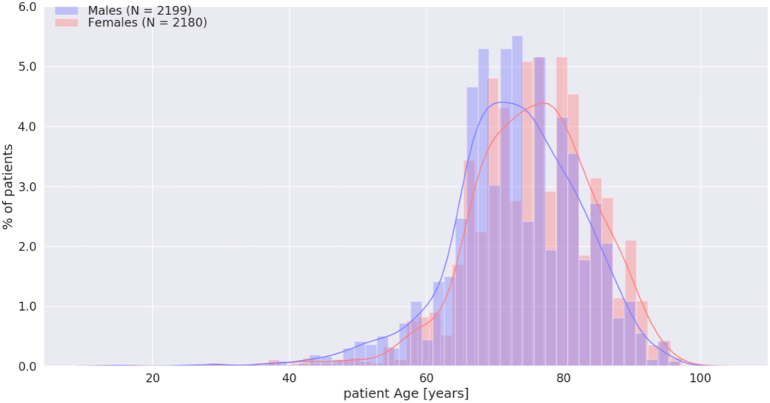
Age distribution of women (red) and men (blue) with AF.

**Fig. 2:**
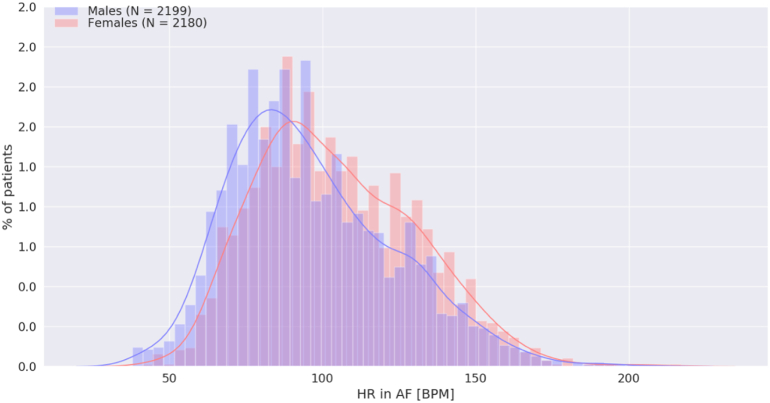
Distribution of the mean heart rate during AF, presented separately for women (red) and men (blue).

**Fig. 3:**
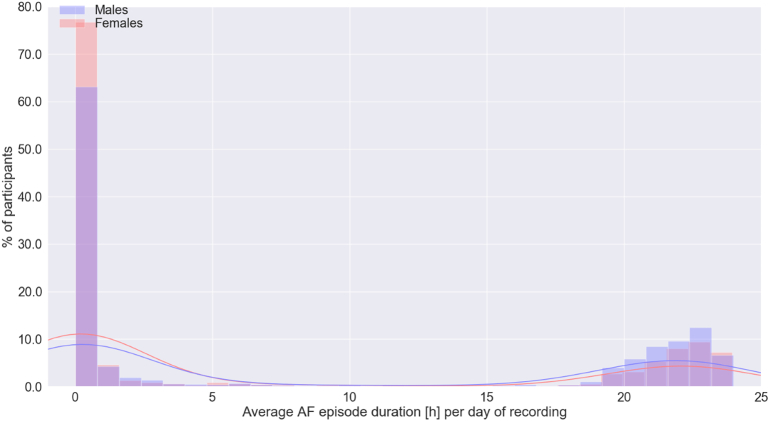
Distribution of the average duration of the AF episodes in hours per day of recording among women (red) and men (blue).

**Fig. 4:**
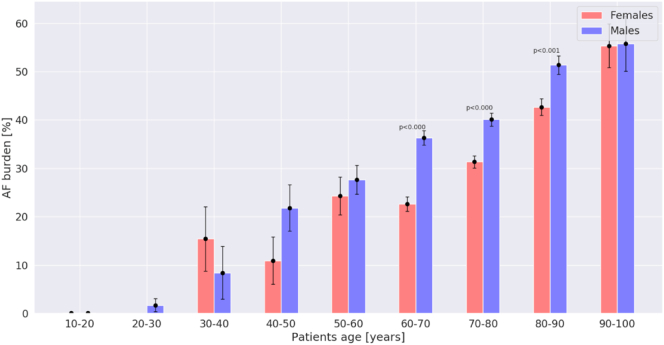
Mean AF burden by percentage in each age group, presented separately for women (red) and men (blue).

Table 2 shows the clinical characteristics of patients with AF stratified by sex. Women with AF were older than men with AF (Fig. 1) Compared with men, women had higher mean heart rate during AF (104.7 ± 26.0 vs 96.7 ± 26.7 bpm, p < 0.001, CI: 6.36 to 9.47) but shorter AF duration (96.2 ± 176.0 vs 121.6 ± 189.9 min, p < 0.001, CI: −36.0 to −14.4), as shown in Fig. 2, Fig. 3. The mean AF burden was lower in women than men in age groups 60–70, 70–80, and 80–90 years old (all p < 0.001), but equivalent in men and women by age 90–100 (Fig. 4). Nonetheless, there was no significant difference in reported symptoms during AF episodes in women and men (46.5 % vs 43.7 %, *X*^*2*^ = 3.50, p = 0.062), although there was a trend toward more symptoms in women. Of note, there were a greater number of women (36.0 %) than men (24.9 %) who reported symptoms in the absence of arrhythmia (*X*^2^ = 63.0, p < 0.001).

**Table 2:** Clinical characteristics of the patients with AF by sex.

Variables	Overall	Women	Men	p value
Patients with AF recorded on 30-day MCT monitors, n (%)	4379	2180 (49.8)	2199 (50.2)	0.968
Age, mean ± SD, years	74.3 ± 9.3	75.6 ± 9.0	73.0 ± 9.5	<0.001
Heart rate in AF, mean ± SD, BPM	100.7 ± 26.6	104.7 ± 26.0	96.7 ± 26.7	<0.001
Duration of AF, mean ± SD (h/day of recording)	7.94 ± 10.2	6.9 ± 9.9	9.0 ± 10.4	<0.001
AF burden, n (%)				<0.001
10–100 %	2289 (52.3)	1038 (47.6)	1251 (56.9)	
1–10 %	1112 (25.4)	582 (26.7)	530 (24.1)	
< 1 %	978 (22.3)	560 (25.7)	418 (19)	
Symptoms reported in AF, n (%)				0.062
Yes	1974 (45.1)	1014 (46.5)	960 (43.7)	
No	2405 (54.9)	1166 (53.5)	1239 (56.3)	
Symptoms reported in the absence of arrhythmia, n (%)				<0.001
Yes	1333 (30.4)	785 (36.0)	548 (24.9)	
No	3046 (69.6)	1395 (64.0)	1651 (75.1)

## 4 Discussion

In this retrospective real-world observational study of patients who received 30-day MCT monitors, we observed several sex differences in presentations with AF. Major findings from our study include the following: (a) a lower proportion of women were diagnosed with AF, (b) women were diagnosed with AF at a later age, (c) women had more rapid mean heart rates during AF, (d) women had a shorter mean AF duration, and (e) women had a lower mean AF burden than men in age groups 60–90 years old. We found no significant difference in the reporting of symptoms during AF in women and men, with a trend toward more symptoms reported in women. However, women were more likely than men to report symptoms unassociated with arrhythmias.

To the best of the authors' knowledge, this is by far the largest real-world observational study examining sex differences in AF with respect to prevalence, burden, heart rate and symptoms during 30-day ambulatory rhythm monitoring using contemporary MCT monitors. Some of these findings are consistent with prior studies, which have shown that women tend to develop AF at a later age than men [12], [13], [14], [15], [16], [17], and that men are at 1.5 to 2.0 times higher risk of developing AF than women [12], [13], [18], [19]. This speaks to the validity of this MCT-derived data for studies of sex-related differences in AF.

The presence of sex-related differences in the electrophysiological characteristics of the pulmonary veins and left atrium may explain a higher prevalence of AF among men [20]. It has also been suggested that lower testosterone levels among older men are associated with an increased risk of AF [21]. Women tend to have later onset of AF. This may be due to effects of endogenous sex hormone such as estrogen which may provide protection against cardiovascular disease and thereby delay the onset of AF [22], [23], [24].

Previous studies have reported that women with AF appear to have higher mean heart rates than men [5], [25], [26], [27]. The present study demonstrates a similar finding, using high-quality MCT data with a beat-to-beat analysis. Nonetheless, the actual mechanism of sex differences in mean heart rates during AF remains unclear and understudied. It has been postulated that lower vagal tone among women might be one of the potential reasons for this observation [5], [25]. Additional factors including emotional stress, sleep quality, burden of co-morbidities, and sex-specific differences in treatment strategies are a few possible mechanistic explanations for this observation.

Similar to data from the Outcomes Registry for Better Informed Treatment of AF (ORBIT-AF) and the Catheter Ablation Versus Antiarrhythmic Drug Therapy for Atrial Fibrillation (CABANA) trials [28], [29], we found that women had lower AF burden and shorter AF duration compared with men. These findings are important, as they may help to explain the observed sex-related disparity in AF management in a real-world setting. For example, women are less likely to be referred to an electrophysiologist or undergo cardioversion or catheter ablation when compared with men [17], [30], [31], [32], [33]. Female sex is an established independent risk factor for thromboembolic events in patients with non-valvular AF [4], [34], [35]. A nationwide cohort study of patients with cardiac implanted electronic devices showed that women with AF >6 h per week was associated with increased risks of stroke, heart failure, and all-cause mortality when compared with men [36]. In spite of this, some studies have shown that women are less likely to receive oral anticoagulation than men [32], [37], although more recent study using a disease-specific registry suggests improvement in this treatment gap [38]. Further investigation is warranted to address potential disparities and gaps in treatment strategies related to management of AF in women versus men.

Although our data did show a trend toward a higher number of women reporting symptoms during AF than men, it was not statistically significant. Contrary to our study, several other studies have demonstrated a significantly greater occurrence of reported symptoms in women compared to men with AF [5], [17], [28], [39]. However, the clinical rhythm during symptoms was not necessarily noted, and symptoms reported during monitoring were not necessarily associated with arrhythmias. In fact, our study did show that women were more likely than men to experience symptoms during sinus rhythm.

So why are women less likely to undergo rhythm control therapy than men? This cannot be explained by a difference in symptoms, as women experience symptoms at least as often as men during AF. More recent trials (EARLY-AF and STOP AF [Sustained Treatment of Paroxysmal Atrial Fibrillation]) have demonstrated the benefit of early rhythm control with respect to long-term outcomes of atrial tachyarrhythmia recurrence, arrhythmia burden, and quality of life [9], [40]. In addition, women as well as men have been shown to have improved rhythm control with AF ablation compared to medical therapy in the CABANA trial [29]. These results highlight the need for further investigation into the reasons for sex differences in therapies to reduce disparities and gaps in care related to AF therapies.

### 4.1 Limitations

The strengths of our study include a large dataset with up to 30-day cardiac rhythm recordings, beat-to-beat analysis, and a large percentage of women to examine for sex differences in AF. Our study has a few important limitations, most of which are related to the nature of the data. Data are limited to epidemiological observations of the sex differences in AF, based on ECG derived parameters. We did not have data on co-morbidities, ethnicity, social factors, laboratory parameters, medications and AF treatments. Hence, we cannot examine potential contributing factors or clinical outcomes related to presentations of AF in this study. Future studies that combine detailed ECG data with clinical parameters, treatment, and outcomes are needed to help identify potential reasons for sex differences in presentation or previously reported disparities in care between men and women.

## 5 Conclusions

Women diagnosed with AF had higher mean heart rates during AF, lower AF burden, and lower AF episode duration compared to men. Women are more likely to have symptoms reported during MCT monitoring, but there were no significant sex differences in symptoms reported during recordings of AF. The results from the present study indicate that factors related to AF burden and duration are a few possible reasons for previously reported sex differences in AF therapies; however, these should be explored in detail in future studies.

## 6 Perspectives

### 6.1 Competency in medical knowledge

In this study, we found that fewer women than men had AF, women were older at the time of diagnosis of AF on monitoring, and women with AF had higher mean heart rate, shorter mean AF duration, and lower mean AF burden than men. There were no sex differences in reported symptoms during AF episodes.

### 6.2 Translational outlook

Sex differences exist with respect to prevalence, age of onset, burden, mean heart rate and duration of AF, with the exception of symptoms during AF. This study shows that previously reported sex differences in AF therapies cannot be explained by symptoms during AF. Results of this study should further prompt researchers to examine reasons for sex differences in AF therapy, including potential reasons related to AF burden and duration.

## Declaration of competing interest

The authors declare the following financial interests/personal relationships which may be considered as potential competing interests:

- Dr. Marek Dziubinski's disclosure: shareholder of Medicalgorithmics/MediLynx
- Dr. Natan Napiorkowski's disclosure: employee of Medicalgorithmics/MediLynx
- Olga Witkowska's disclosure: employee of Medicalgorithmics/MediLynx
- Magdalena E Slusarczyk's disclosure: employee of Medicalgorithmics/MediLynx
- Dr. Jeff S. Healey's disclosure: research funding from Boston Scientific, Medtronic, Abbott, BMS/Pfizer with consulting fees from Boston Scientific, BMS/Pfizer and Bayer
- Dr. Andrea M. Russo's disclosure: research trials, funding to hospital (Boston Scientific, Kestra, Medilynx); research steering committee (Boston Scientific, Medtronic); consultant (Atricure, Biosense Webster, Boston Scientific, Medtronic)
- All other authors have no conflicts of interest to disclose.
